# Tooth loss in complying and non-complying periodontitis patients with different periodontal risk levels during supportive periodontal care

**DOI:** 10.1007/s00784-021-03895-8

**Published:** 2021-03-24

**Authors:** Roberto Farina, Anna Simonelli, Andrea Baraldi, Mattia Pramstraller, Luigi Minenna, Luca Toselli, Elisa Maietti, Leonardo Trombelli

**Affiliations:** 1 Research Centre for the Study of Periodontal and Peri-Implant Diseases, University of Ferrara, Corso Giovecca 203, 44121 Ferrara, Italy; 2 Operative Unit of Dentistry, Azienda Unità Sanitaria Locale (AUSL) of Ferrara, Ferrara, Italy; 3 Department of Medical and Surgical Sciences, University of Bologna, Bologna, Italy; 4 Center for Clinical Epidemiology, University of Ferrara, Ferrara, Italy

**Keywords:** Supportive periodontal therapy, Periodontitis, Tooth loss, Prognosis, Risk, Risk assessment

## Abstract

**Objectives:**

To evaluate yearly tooth loss rate (TLR) in periodontitis patients with different periodontal risk levels who had complied or not complied with supportive periodontal care (SPC).

**Materials and methods:**

Data from 168 periodontitis patients enrolled in a SPC program based on a 3-month suggested recall interval for at least 3.5 years were analyzed. For patients with a mean recall interval within 2–4 months (“compliers”) or > 4 months (“non-compliers”) with different PerioRisk levels (Trombelli et al. 2009), TLR (irrespective of the cause for tooth loss) was calculated. TLR values were considered in relation to meaningful TLR benchmarks from the literature for periodontitis patients either under SPC (0.15 teeth/year; positive benchmark) or irregularly complying with SPC (0.36 teeth/year; negative benchmark).

**Results:**

In both compliers and non-compliers, TLR was significantly below or similar to the positive benchmark in PerioRisk level 3 (0.08 and 0.03 teeth/year, respectively) and PerioRisk level 4 (0.12 and 0.18 teeth/year, respectively). Although marked and clinically relevant in non-compliers, the difference between TLR of compliers (0.32 teeth/year) and non-compliers (0.52 teeth/year) with PerioRisk level 5 and the negative benchmark was not significant.

**Conclusion:**

A SPC protocol based on a 3- to 6-month recall interval may effectively limit long-term tooth loss in periodontitis patients with PerioRisk levels 3 and 4. A fully complied 3-month SPC protocol seems ineffective when applied to PerioRisk level 5 patients.

**Clinical relevance:**

PerioRisk seems to represent a valid tool to inform the SPC recall interval as well as the intensity of active treatment prior to SPC enrollment.

## Introduction

At completion of active periodontal therapy, periodontitis patients present a varying residual risk for disease recurrence/progression that can be managed with supportive periodontal care (SPC). SPC consists of a series of preventive and therapeutic interventions which are performed/administered professionally at a regular interval. SPC sessions incorporate assessment of periodontal and general health, motivation to self-performed oral hygiene and risk factor control (thus requiring patient contribution in terms of behavioral change), professional mechanical plaque removal (PMPR), and sub-gingival instrumentation of residual pockets [1]. In patients previously treated for varying severity of periodontitis, a SPC program based on a regular (1–6 months) recall interval has been associated with weighted mean yearly tooth loss rates (TLR) of 0.15 and 0.09 for studies with a follow-up of 5 years and 12–14 years, respectively [2]. Although a routine SPC regimen has robustly shown essential to limit tooth loss in treated periodontitis patients [1–4], specific patient groups still manifest high tooth loss rates over the maintenance phase [5–9].

Several periodontitis-related factors have been shown to contribute between-subject variability in tooth loss during SPC. Following active therapy, residual exposure to modifiable risk factors such as smoking [10–12] and diabetes [13–15] and the residual diseased sites following active treatment [4, 16, 17] were shown to negatively impact tooth loss during SPC. For instance, we have previously demonstrated that an increasing rate of tooth loss was positively associated with the proportion of residual bleeding pockets at SPC enrollment [4]. Also, patient adherence with the suggested SPC program (i.e., the patient compliance) may frequently be less than optimal, with proportions of non-compliers up to 64.4% [18], and was associated with higher rates of tooth loss [19–22]. Variability in patient response to SPC raises the clinically relevant issue of the appropriateness of a specific protocol for the individual patient, including session intervals, intervention modalities, and strategies to optimize patient compliance [4].

There is currently a wide consensus on the fact that the evaluation of the patient risk profile with validated periodontal risk assessment tools may represent a promising approach to predict disease progression in terms of tooth/bone loss [23, 24]. In this respect, it has been previously shown that the provision of the same SPC protocol (in terms of clinical procedures and annual frequency of SPC sessions) in groups with different periodontal risk levels at completion of active therapy resulted in unfavorable outcomes in the highest risk category [9]. Higher tooth loss has been consistently observed in patients with a high-risk profile despite a stringent recall program [7, 9, 25]. Overall, these findings suggest our current inability to effectively match the adopted secondary preventive protocol with the individual need, thus resulting in under-provision of care to some individuals and over-provision to others. This can result in increased burden of disease, unwanted side effects, and sub-optimal allocation of resources [3].

A study conducted by Matuliene et al. (2010) [25] suggested a potential association among tooth loss, periodontal risk level, and patient compliance with suggested SPC protocol. The aim of the present study was to evaluate tooth loss in periodontitis patients with different periodontal risk levels who had either complied or not complied with SPC. The rationale to separately analyze cohorts with different levels of compliance (resulting in different intervals between recall sessions) was to (i) provide better insight into the appropriateness of a 3-month stringent and fully complied SPC protocol for patients with different periodontal risk levels and (ii) determine whether and to what extent the lack of patient compliance may affect SPC under different periodontal risk level conditions.

## Materials and methods

### Experimental design and ethical aspects

The present study is a retrospective analysis of de-identified data derived from the record charts of patients seeking care at the Research Centre for the Study of Periodontal and Peri-implant Diseases, University of Ferrara, Ferrara, Italy; two private dental offices in Ferrara, Italy; and one private dental office in Padova, Italy. The study protocol was approved by the ethical committee of Area Vasta Emilia Centro, Regione Emilia-Romagna (CE-AVEC) (protocol number: 58/2020/Oss/UniFe). All the clinical procedures were performed in full accordance with the Declaration of Helsinki and the Good Clinical Practice Guidelines (GCPs). Each patient included in the present analysis had previously given a written informed consent to periodontal treatments.

Patient selection was based on inclusion and exclusion criteria (see “Study population”) and the availability of specific data (see “Study parameters”) related to a visit performed ≤ 12 months following the completion of active periodontal therapy (i.e., the *baseline*) and the first visit among those performed ≥ 3.5 years from baseline (i.e., the *follow-up*).

### Study population

Patients were included in the study if positive for all the following inclusion criteria:

- ≥ 18 years (at initial visit);
- Diagnosis of periodontitis according to the classification system in use at the time [26, 27];
- Undergoing active periodontal therapy, consisting of non-surgical instrumentation (single or multiple sessions of supra- and sub-gingival instrumentation with or without additional use of local/systemic antimicrobials) eventually followed by one or more sessions of periodontal surgery;
- Enrolled in a SPC program with a 3-month suggested interval between SPC sessions;
- Participation in the SPC program for ≥ 3.5 years (irrespectively of the level of adherence to the suggested SPC recall interval);
- Availability of data related to the medical and dental history as well as the clinical and radiographic documentation (see “Study parameters” for details) related to baseline and follow-up.

Patients were excluded from the study if positive for one or more of the following conditions with a documented impact on periodontal conditions and/or periodontal treatment outcomes: pregnancy, genetic diseases (e.g., Down’s syndrome), immune system diseases (e.g., HIV), hematologic disorders with a quantitative or qualitative deficit of leucocytes, and physical and mental handicaps interfering with self-performed oral hygiene procedures.

### Clinical procedures during SPC

Each SPC session included [4]:

(i) Review and update of patient medical and dental history;
(ii) Clinical examination, including evaluation of periodontal and peri-implant tissue conditions. In particular, probing depth (PD) and bleeding on probing (BoP) were evaluated at each SPC session and were recorded yearly;
(iii) Assessment of the patient’s oral hygiene performance. A plaque disclosing agent was used to facilitate patient motivation and professional plaque removal. The Plaque Index was not routinely recorded;
(iv) Assessment of modifiable risk factors;
(v) Professional, mechanical removal of supra- and sub-gingival plaque and calculus. While supra- or juxta-gingival instrumentation was performed at all sites, sub-gingival instrumentation was restricted to sites with PD ≥ 4 mm;
(vi) Behavior modification (such as oral hygiene reinstruction, compliance with suggested periodontal maintenance intervals, and counseling on control of risk factors);
(vii) Delivery of antimicrobial agents with documented efficacy at sites with PD ≥ 6 mm at operator discretion.

### Study parameters

#### Demographic, smoking status, and diabetic status

The following data related to the baseline visit were derived from each clinical record chart:

- Age (years);
- Gender;
- Smoking status (current smoker, irrespective of the number of cigarettes smoked per day and years of smoking exposure; former smoker, irrespective of the time elapsed from quitting smoking; never smoked);
- Number of cigarettes per day;
- Diabetic status (diabetic, non-diabetic);
- Metabolic control of diabetes (plasma level of HbA1c).

#### Compliance to SPC

The number of attended sessions of supra- and sub-gingival plaque removal during the observation period (i.e., between baseline and follow-up) was extracted from the clinical record charts of each patient. The mean interval between SPC sessions was derived as the length of the observation period (in months)/number of attended SPC sessions. Patients were considered “compliers” or “non-compliers” if their mean interval between SPC sessions fell within the range of 2–4 months or was > 4 months, respectively.

#### Clinical parameters

At baseline and follow-up, the number of teeth present was recorded from the clinical periodontal chart of each patient.

Also, the following clinical parameters related to baseline were extracted:

- Number of sites with PD ≥ 5 mm, where PD had been assessed using a manual periodontal probe (CP12) at mesio-buccal, buccal, disto-buccal, mesiolingual, lingual, and disto-lingual for each tooth including fully erupted third molars;
- BoP score, calculated as the percentage proportion of sites positive to BoP after probe insertion up to the bottom of the sulcus/pocket.

#### Radiographic parameters

For each patient, a full-mouth set of periapical radiographs was taken at baseline by means of analog films (Kodak, Rochester, NY, USA). Radiographs were digitized at 600 dpi, and linear radiographic measurements were performed by a single examiner using dedicated software (NIS Elements^TM^; Nikon Instruments S.P.A. Campi Bisenzio, Firenze, Italy). Radiographic assessments were preceded by a calibration phase, performed on radiographs of patients not included in the study. The evaluation of intra-examiner agreement revealed good consistency of radiographic measurements (intra-class correlation coefficient ≥ 0.70). At the mesial and distal aspect of each tooth, the distance (in mm) between the cementum–enamel junction (CEJ) and the bone crest (BC) was measured (CEJ-BC) with a digital caliper. At sites where the CEJ could not be identified due to the presence of restorations, the distance between the apical margin of the restoration and BC was measured. Measurements were rounded to the nearest 0.1 mm. All sites where the CEJ, the restoration margin, and the BC profile could not be identified were excluded from the analysis.

#### Periodontitis staging and grading

For the purpose of the present study, periodontitis diagnosis received at initial visit according to the classification system in use at the time [26, 27] was reconsidered according to the staging and grading system as proposed in the 2017 World Workshop for the Classification of Periodontal and Peri-implant Diseases and Conditions [28, 29].

#### Periodontal risk assessment

Based on baseline data, the patient risk assessment was performed according to the PerioRisk, as previously proposed [30, 31] and validated during SPC [9, 24]. PerioRisk is based upon five parameters derived from a patient’s medical history and clinical recordings (smoking, diabetes, number of sites with PD ≥ 5 mm, BoP score, and the number of teeth showing a bone loss ≥ 4 mm per age of the patient). Risk calculation is described in details in Tables 1, 2, 3, 4, 5, and 6. Briefly, each parameter is allocated a “parameter score” ranging from 0 to 8 for one parameter (i.e., number of teeth showing a bone loss ≥ 4 mm per age of the patient) and from 0 to 4 for the other parameters according to predefined tables (Tables 1, 2, 3, 4, and 5). The algebraic sum of the parameter scores is then calculated, producing a “PerioRisk level” ranging from 1 (lowest risk) to 5 (highest risk) (Table 6). Also, the tool provides granular information on the “PerioRisk profile,” i.e., the contribution of each parameter score to the PerioRisk level.

**Table 1:** PerioRisk method [30]: generation of the parameter score related to smoking

Smoking status	Parameter score
Never smoked	0
Former smoker	1
1–9 cigarettes per day	2
10–19 cigarettes per day	3
≥ 20 cigarettes per day	4

**Table 2:** PerioRisk method [30]: generation of the parameter score related to diabetes

Diabetic status	Parameter score
Non-diabetic	0
Controlled diabetic (sieric HbA1c < 7.0%)	2
Poorly controlled diabetic (sieric HbA1c ≥ 7.0%)	4

**Table 3:** PerioRisk method [30]: generation of the parameter score related to the number of sites with probing depth ≥ 5 mm

Number of sites with probing depth ≥ 5 mm	Parameter score
0–1	0
2–4	1
5–7	2
8–10	3
> 10	4

**Table 4:** PerioRisk method [30]: generation of the parameter score related to the Bleeding on Probing Score

Bleeding on Probing Score (%)	Parameter score
0–5%	0
6–16%	1
17–24%	2
25–36%	3
> 36%	4

**Table 5:** PerioRisk method [30]: generation of the parameter score related to the extent of bone loss/age

		Bone loss (no. of teeth with CEJ-BC ≥ 4 mm)				
		0	1–3	4–6	7–10	> 10
Age (years)	0–25	0	8	8	8	8
	26–40	0	6	6	8	8
	41–50	0	4	4	6	8
	51–65	0	2	4	6	8
	> 65	0	0	2	4	6

**Table 6:** PerioRisk method [30]: determination of the periodontal risk level. The parameter scores obtained from Tables 1, 2, 3, 4, and 5 are added, and the sum (in parenthesis) is referred to a risk level ranging from 1 (low risk) to 5 (high risk)

Risk level: 1 Low risk	Risk level: 2 Low–medium risk	Risk level: 3 Medium risk	Risk level: 4 Medium–high risk	Risk level: 5 High risk
(0–2)	(3–5)	(6–8)	(9–14)	(15–24)

### Statistical analysis

The patient was the statistical unit for the analysis. Data were expressed as mean ± standard deviation (SD) or median and inter-quartile (IQ) range in case of strongly asymmetric distributions. Comparisons among patients with different PerioRisk levels with regard to baseline characteristics were conducted using Kruskal–Wallis test and Pearson’s chi-squared test for continuous and categorical variables, respectively.

For each patient, TLR was calculated as the ratio between the number of teeth lost during the observation period and the duration of the observation period (in years) and represented the primary outcome variable of the study. Mean TLR is a measure of incidence and was expressed as the number of teeth lost per person-year.

Two meaningful values were identified from the literature: (i) 0.15 teeth/year (positive benchmark) as reported for periodontitis patients under SPC in a systematic review [2] and (ii) 0.36 teeth/year as reported for treated, moderate–severe periodontitis patients irregularly complying with a SPC program (negative benchmark) [32].

Comparison in TLR between groups and with respect to positive and negative benchmarks was performed using negative binomial regression analysis, with TLR as dependent variable. Regression results were reported as mean TLR and 95% confidence interval (95%CI). A 95%CI that includes the benchmark indicates no significant difference. A multiple model was estimated in order to assess both PerioRisk level and compliance net effect.

The level of statistical significance was set at *p* < 0.05. Analyses were performed using Stata statistical software (StataCorp 2013. StataCorp LP, College Station, TX).

## Results

### Study population

One hundred eighty-three patients were eligible for the study. The number of patients with PerioRisk levels 1, 2, 3, 4, and 5 was 9, 6, 37, 104, and 27, respectively. Due to the limited numerosity of groups with PerioRisk levels 1 and 2, the present analysis was restricted to PerioRisk levels 3, 4, and 5. Patient distribution according to periodontitis stage and grade is reported in Table 7. Baseline patient characteristics in the entire study population as well as within PerioRisk levels 3–5 are summarized in Table 8. The median duration of the observation period was 5.0 years and ranged between 3.5 and 15 years (Table 8).

**Table 7:** Patient distribution according to periodontitis stage, grade, and extent, as determined according to the 2017 classification system [28, 29]

		Periodontitis stage and extent							
		Stage 1		Stage 2		Stage 3		Stage 4	
		Localized or molar/incisor pattern	Generalized	Localized or molar/incisor pattern	Generalized	Localized or molar/incisor pattern	Generalized	Localized or molar/incisor pattern	Generalized
Periodontitis grade	A	0	0	1	0	3	16	0	1
	B	0	0	4	0	10	68	0	5
	C	0	0	3	0	5	41	0	11

**Table 8:** Baseline patient characteristics and duration of SPC in the entire study population as well within each PerioRisk level [30]

	Entire study population	PerioRisk level = 3	PerioRisk level = 4	PerioRisk level = 5	*p* value for inter-group comparisons
	(*n* = 168)	(*n* = 37)	(*n* = 104)	(*n* = 27)	
Age (years)					0.635
Mean (± SD)	47.0 (± 10.3)	48.3 (± 7.8)	46.8 (± 11.3)	45.7 (± 9.7)	
Gender					0.540
Males, *n* (%)	72 (42.9)	14 (37.8)	44 (42.3)	14 (51.9)	
Smoking					< 0.001
Never smoked, *n* (%) Former smokers, *n* (%) Smokers, *n* (%) 1–9 sig/die, *n* (%) 10–19 sig/die, *n* (%) ≥ 20 sig/die, *n* (%)	94 (56.0) 21 (12.5) 53 (31.5) 18 (10.7) 26 (15.5) 9 (5.3)	25 (67.6) 6 (16.2) 6 (16.2) 5 (13.5) 1 (2.7) 0	63 (60.6) 12 (11.5) 29 (27.9) 8 (7.7) 17 (16.3) 4 (3.8)	6 (22.2) 3 (11.1) 18 (66.7) 5 (18.5) 8 (29.6) 5 (18.5)	
Diabetes					0.790
*n* (%)	4 (2.4)	1 (2.7)	2 (1.9)	1 (3.7)	
Number of sites with PD ≥ 5 mm					< 0.001
Median [IQ range] 0–1, *n* (%) 2–4, *n* (%) 5–7, *n* (%) 8–10, *n* (%) > 10, *n* (%)	7 [4–16] 27 (16.1) 26 (15.5) 36 (21.4) 17 (10.1) 62 (36.9)	5 [1–7] 12 (32.4) 5 (13.5) 12 (32.4) 1 (2.7) 7 (18.9)	6 [3–13] 15 (14.4) 20 (19.2) 24 (23.1) 13 (12.5) 32 (30.8)	20 [12–38] 0 1 (3.7) 0 3 (11.1) 23 (85.2)	
BoP score (%)					< 0.001
Median [IQ range] 0–5, *n* (%) 6–16, *n* (%) 17–24, *n* (%) 25–36, *n* (%) < 36, *n* (%)	8 [1.5–18.5] 71 (42.3) 43 (25.6) 33 (19.6) 11 (6.5) 10 (6.0)	5 [0–13] 20 (54.1) 9 (24.3) 6 (16.2) 1 (2.7) 1 (2.7)	7 [1–17] 48 (46.1) 28 (26.9) 20 (19.2) 4 (3.9) 4 (3.9)	20 [11–35] 3 (11.1) 6 (22.2) 7 (25.9) 6 (22.2) 5 (18.5)	
No. of teeth with CEJ–BC≥ 4 mm					< 0.001
Median [IQ range] 0, *n* (%) 1–3, *n* (%) 4–6, *n* (%) 7–10, *n* (%) > 10, *n* (%)	12 [6–18] 8 (4.8) 18 (10.7) 17 (10.1) 33 (19.6) 92 (54.8)	5 [2–9] 6 (16.2) 8 (21.6) 8 (21.6) 10 (27.0) 5 (13.5)	14 [8–18] 2 (1.9) 10 (9.6) 9 (8.7) 19 (18.3) 64 (61.5)	18 [13–22] 0 0 0 4 (14.8) 23 (85.2)	
Number of teeth analyzed					0.310
Mean (± SD)	25.3 (± 3.3)	25.0 (± 2.8)	25.1 (± 3.4)	26.2 (± 3.6)	
Duration of SPC (years) Median [IQ range] Min–max	4 [4–5] 3.5–15	4 [3.5–5] 3.5–10	4 [4–6] 3.5–15	4 [3.5–5] 3.5–9	0.377

Patient distribution according to mean SPC recall interval is reported in Table 9. Compliers (82.7% of the study population) had a mean interval between SPC sessions of 3.3 ± 0.5 months, whereas non-compliers (17.3% of the study population) had a mean interval between SPC sessions of 6.3 ±1.5 months. The proportion of compliers in PerioRisk levels 3, 4, and 5 was 78%, 86%, and 78%, respectively (*p* = 0.462).

**Table 9:** Patient distribution according to mean SPC recall interval^*^

	Mean SPC recall interval (months)	Entire study population (*n* = 168)	PerioRisk level		
			3 (*n* = 37)	4 (*n* = 104)	5 (*n* = 27)
Compliers	2	4	2	2	0
	3	93	21	56	16
	4	42	6	31	5
Non-compliers	5	2	1	1	0
	6	25	7	13	5
	12	2	0	1	1

^*^Mean SPC recall interval was derived as the length of the observation period (in months)/number of attended SPC sessions. Patients were considered “compliers” or “non-compliers” if their mean interval between SPC sessions fell within the range of 2–4 months or was > 4 months, respectively

One hundred twenty-eight teeth were lost during the observation period, with a mean TLR of 0.15.

### Tooth loss in patients with different levels of either periodontal risk or compliance to SPC

Data on tooth loss within PerioRisk levels 3, 4, and 5 is reported in Table 10. Mean TLR was 0.06, 0.13, and 0.37 in patients with PerioRisk levels 3, 4, and 5, respectively (*p* < 0.001). Mean TLR in compliers and non-compliers was 0.14 (95%CI: 0.10–0.18) and 0.21 (95%CI: 0.09–0.33), respectively (*p* = 0.200).

**Table 10:** Tooth loss in patients with different PerioRisk level [30]

PerioRisk level	Number	*n* (%) of patients losing at least 1 tooth during the observation period	Total no. of teeth lost within the group during the observation period	No. of teeth lost per patient during the observation period	Tooth loss rate (TLR) (95%CI)
3	37	9 (24.3%)	11	0.3 (± 0.6)	0.06 (0.02–0.11)
4	104	40 (38.5%)	71	0.7 (± 1.2)	0.13 (0.09–0.17)
5	27	19 (70.4%)	46	1.7 (± 2.0)	0.37 (0.20–0.53)
*p*-value		0.001	-	< 0.001	< 0.001

**Fig. 1:**
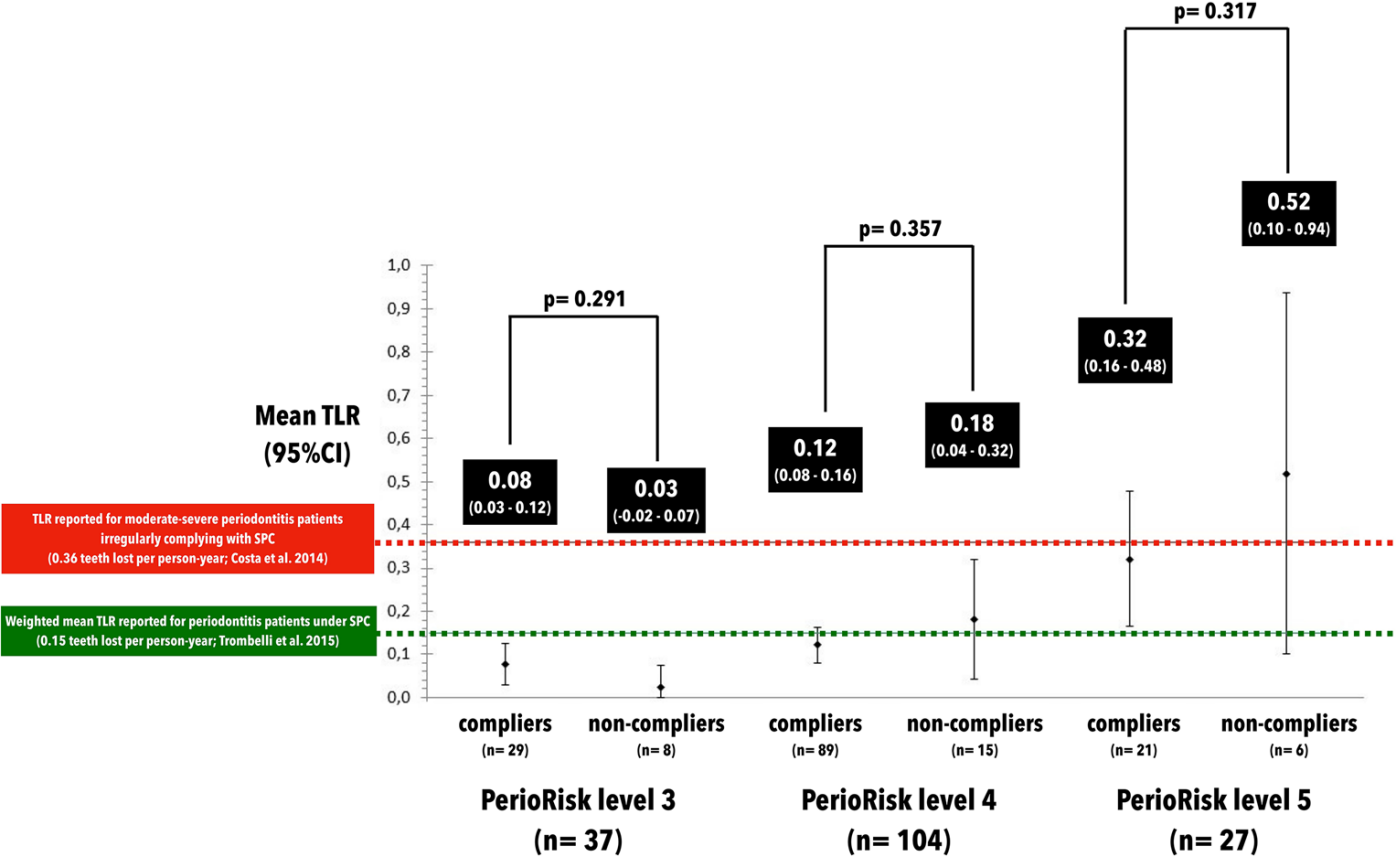
TLR (illustrated as mean and 95%CI) in patients with different mean frequency of SPC within each PerioRisk level (calculated according to PerioRisk) [30]

TLR for compliers and non-compliers within each PerioRisk level is shown in Fig. 1.

In patients with PerioRisk level 3, mean TLR was significantly below the positive benchmark for both compliers (0.08, 95%CI: 0.03–0.12) and non-compliers (0.03, 95%CI: −0.02–0.07). No significant difference in TLR was found between the two groups (*p* = 0.291). Within PerioRisk level 3, the proportion of patients losing at least 1 tooth during the observation period was 28% and 13% for compliers and non-compliers, respectively, with no significant inter-group difference (*p* = 0.649).

In patients with PerioRisk level 4, TLR was similar to the positive benchmark and significantly lower than the negative benchmark. Mean TLR was 0.12 (95%CI: 0.08–0.16) in compliers and 0.18 (95%CI: 0.04–0.32) in non-compliers, with no significant difference between groups (*p* = 0.357). Within PerioRisk level 4, the proportion of patients losing at least 1 tooth during the observation period was 36% and 53% for compliant and non-compliant patients, respectively (*p* = 0.254).

In patients with PerioRisk level 5, mean TLR in compliers (0.32, 95%CI: 0.16–0.48) was significantly higher than the positive benchmark and similar to the negative benchmark. Although not reaching statistical significance, TLR of non-compliers (0.52, 95%CI: 0.10–0.94) was markedly higher compared to either the TLR of compliers or the negative benchmark. Within PerioRisk level 5, the proportion of patients losing at least 1 tooth during the observation period was 67% and 83% for compliers and non-compliers, respectively (*p* = 0.633).

When a multiple model was implemented using TLR as dependent variables and PerioRisk and compliance as dependent variables, only PerioRisk significantly explained TLR (*p* < 0.001). Significant differences in TLR were found between levels 3 and 5 (*p* < 0.001) and levels 4 and 5 (*p* < 0.001), while the difference between 3 and 4 was of borderline significance (*p* = 0.053).

## Discussion

The aim of the present study was to evaluate tooth loss in periodontitis patients with different periodontal risk levels who had either complied or not complied with SPC. One hundred and sixty-eight periodontitis patients enrolled in a SPC program based on a 3-month suggested interval between SPC sessions for at least 3.5 years after the completion of active therapy were included in the present analysis, and de-identified data were retrospectively derived from their record charts. Patients were stratified according to their baseline risk level, as calculated according to PerioRisk [30]. Within each available PerioRisk level (i.e., moderate risk, PerioRisk level 3; moderate–high risk, PerioRisk level 4; and high risk, PerioRisk level 5), TLR was calculated for compliers and non-compliers. In order to determine whether compliers/non-compliers are associated with either a successful or a non-successful SPC program, TLR was compared to a positive [2] and a negative [32] benchmark, respectively, within each PerioRisk level.

In our material, three cohorts of patients with different risk levels (3, 4, and 5) as calculated according to PerioRisk [30] were retrospectively identified. Consistent with previous studies [9], TLR was significantly associated with PerioRisk level, being 0.06 teeth/year for risk 3, 0.13 teeth/year for risk 4, and 0.37 teeth/year for risk 5. Moreover, the proportion of patients losing at least 1 tooth during the observation period increased at an increasing PerioRisk level. Also, the influence of PerioRisk level on TLR was evident irrespective of patient compliance. Overall, these findings further reinforce the association of the PerioRisk level and tooth loss during SPC [9]. In a recent study where four periodontal risk assessment tools were compared for their prognostic performance in terms of periodontal-related tooth loss, PerioRisk showed the best discrimination and model fit [24].

Several definitions of non-compliance to SPC have been proposed in the literature, with a variable impact on the risk ratio for tooth loss depending on the level of stringency (strict or range) to define a non-compliant patient [19]. In the present study, compliers or non-compliers were identified as two mutually exclusive patient categories based on their level of adherence with the suggested 3-month interval between SPC sessions. For non-compliers, mean SPC interval resulted in 6.3 months, due to the great majority of patients (86.2%) attending a mean 6-month recall interval. TLR was 0.14 and 0.21 teeth/year in compliers and non-compliers, respectively. Although not statistically significant, this difference may be considered of clinical relevance.

This retrospective study aimed at evaluating the impact of a 3-month stringent and fully complied SPC protocol on tooth loss in patients with different periodontal risk levels. In compliers, TLR was below or similar to the positive benchmark for PerioRisk levels 3 and 4, respectively. By contrast, TLR in compliers for PerioRisk level 5 (0.32 teeth/year) was significantly higher than the positive benchmark (0.15 teeth/year) and similar to the negative benchmark (0.36 teeth/year). These findings suggest that a 3-month SPC protocol is effective in limiting long-term tooth loss in periodontitis patients showing a PerioRisk level of 3 or 4 whereas appears inefficient when applied to patients with the highest PerioRisk level. When considering that high-risk patients are also those showing less adherence to the SPC program in the long term [25, 33–35], the adoption of a SPC program based on a more stringent (i.e., < 3-month) interval between sessions might represent a weak option to enhance SPC efficacy over time. Considering that patients with PerioRisk level 5 are significantly different in terms of smoking and disease-associated markers (Table 8), a more efficient treat-to-target approach based on smoking cessation program [36–38] as well as a more intense treatment of pockets and periodontal inflammation may be recommended prior to SPC enrollment in individuals at the highest risk level, particularly if not complying with the suggested SPC recall interval. This recommendation is also supported by previous data showing that high (≥ 30%) BoP score [8] and increasing proportions of bleeding pockets [4] at re-evaluation following active therapy were positively associated with greater tooth loss during SPC.

Interestingly, in non-compliers with PerioRisk levels 3 and 4, TLR was still below or close to, respectively, the positive benchmark of 0.15 teeth/year. These findings suggest that in patients showing a PerioRisk level 3 or 4 at completion of active therapy, a recall interval of up to 6 months might be compatible with a limited extent of tooth loss in the long-term. In this respect, although more frequent SPC recall visits were associated with fewer teeth extracted in some studies [39], other reports showed no statistically significant differences in tooth loss in cohorts with SPC intervals of less than or more than 6 months [40, 41]. Within PerioRisk level 5, TLR of non-compliers (0.52 teeth/year) was evidently higher than both the TLR of compliers (0.32 teeth/year) and the negative benchmark (0.36 teeth/year). Although the difference did not reach statistical significance (probably due to the limited size of the non-compliers sample and the high data dispersion within the latter), this finding seems to indicate that the lack of adherence with a SPC program based on a 3–4-month interval may further accelerate the rate of tooth loss in patients at high risk.

The findings from the present study must be considered in light of some limitations, which are mainly due to the retrospective nature of the study. First, it was not possible to retrieve information on the causes for tooth loss or extraction. In absence of this information, it is uncertain whether tooth loss may represent a true indicator of periodontitis progression. Periodontal disease, however, was often reported as the main reason for tooth loss in several prospective [32, 42–44] and retrospective [5, 6, 38, 45–47] studies on the efficacy of periodontal maintenance programs in patients treated for periodontitis. It should also be stressed that some patient groups (e.g., non-compliers with PerioRisk levels 3 and 5) presented with a limited numerosity. Sample size limitations unavoidably limit the strength of the present observations and call for further studies on larger samples to validate our findings.

In conclusion, the results of the present study indicate that a SPC protocol based on a 3- to 6-month recall interval may effectively limit long-term tooth loss in periodontitis patients with PerioRisk levels 3 and 4. By contrast, even a fully complied 3-month SPC protocol seems to be ineffective when applied to patients with PerioRisk level 5.
